# Out-of-pocket expenditures for HIV in the Dominican Republic: findings from a community-based participatory survey

**DOI:** 10.26633/RPSP.2019.56

**Published:** 2019-06-07

**Authors:** Claire Chaumont, Carlo Oliveira, Enrique Chavez, Jaime Valencia, Pablo Villalobos Dintrans

**Affiliations:** 1 Harvard T.H. Chan School of Public Health Harvard T.H. Chan School of Public Health BostonMassachusetts United States of America Harvard T.H. Chan School of Public Health, Boston, Massachusetts, United States of America.; 2 International Treatment Preparedness Coalition International Treatment Preparedness Coalition New YorkNew York United States of America. International Treatment Preparedness Coalition, New York, New York, United States of America.; 3 AID FOR AIDS AID FOR AIDS New YorkNew York United States of America. AID FOR AIDS, New York, New York, United States of America.; 4 Universidad de Santiago Universidad de Santiago Santiago Chile Universidad de Santiago, Santiago, Chile.

**Keywords:** HIV, health care costs, health expenditures, community-based participatory research, Dominican Republic, VIH, costos de la atención en salud, gastos en salud, investigación participativa basada en la comunidad, República Dominicana, HIV, custos de cuidados de saúde, gastos em saúde, pesquisa participativa baseada na comunidade, República Dominicana

## Abstract

**Objectives.:**

The aim of this study was to estimate out-of-pocket expenditures incurred by individuals with HIV in the Dominican Republic. The study utilized different definitions and components for these expenditures and differentiated the results by wage ranges.

**Methods.:**

Data was obtained from an in-person survey of people living with HIV. The study was developed and implemented in collaboration with Dominican grassroots organizations and networks of people with HIV, through a process of community-based participatory research.

**Results.:**

The mean HIV-related expenditure reported by individuals in the sample in the last six months prior to the survey was in US$ 181; 15.4% of this total was spent for transportation and housing and costs to access the HIV facility. The mean expenditure reported by individuals for their current visit to an HIV center was US$ 10. These out-of-pocket expenditures exhibited regressivity, with lower-wage patients spending proportionally more to receive care. The results highlight the importance of considering other resources required to access treatment, such as lodging expenses and the time needed to travel to an HIV center and then to wait to be seen by a care provider.

**Conclusions.:**

There should be more focus on expenditures made directly by people with HIV in the Dominican Republic so that these payments do not become a barrier to accessing health care. Using a community-based participatory design can ensure that such data can be leveraged to address the specific barriers to care that are faced by individuals with HIV.

## RESUMEN

**Objetivos.:**

El objetivo de este estudio fue estimar los gastos directos de bolsillo que enfrentan las personas con VIH en la República Dominicana, utilizando diferentes definiciones y componentes para estos gastos y desglosando los resultados por rangos salariales.

**Métodos.:**

Los datos se obtuvieron a partir de una encuesta presencial a personas con infección por el VIH. El estudio se llevó a cabo en colaboración con organizaciones comunitarias locales y redes de personas con infección por el VIH, mediante un proceso de investigación participativa comunitaria.

**Resultados.:**

El gasto directo promedio en los seis meses anteriores a la encuesta para las personas con VIH que conformaban la muestra fue de US$ 181; el 15,4% del gasto directo total se dedicó a transporte, vivienda y otros costos para acceder al establecimiento de salud. El gasto promedio informado por las personas para su cita actual en el establecimiento de salud fue de US$ 10. El gasto directo presentó regresividad: los pacientes de salario inferior gastaban proporcionalmente más para recibir atención. Los resultados destacan la importancia de considerar otros recursos necesarios para obtener acceso al tratamiento, como los gastos de alojamiento y el tiempo invertido en desplazarse a un centro de atención del VIH y esperar a ser atendido por un prestador de atención.

**Conclusiones.:**

Es necesario centrar más la atención en el gasto directo de las personas con VIH en la República Dominicana, para que estos costos no se conviertan en un obstáculo al acceso a la atención de salud. Un enfoque participativo comunitario propiciaría el uso de estos datos para abordar los obstáculos específicos que enfrentan las personas con VIH a la hora de obtener atención.

## RESUMO

**Objetivos.:**

Estimar as despesas desembolsadas por pessoas com HIV na República Dominicana segundo diferentes definições e itens para estas despesas e a diferenciação dos resultados por faixas salariais.

**Métodos.:**

Os dados foram obtidos em uma pesquisa presencial com pessoas com HIV. O estudo foi elaborado e executado em colaboração com organizações locais dominicanas e redes de pessoas com HIV por meio de um processo da pesquisa participativa baseada na comunidade.

**Resultados.:**

A despesa média relacionada ao HIV nos seis meses anteriores à pesquisa informada pelos participantes na amostra estudada foi de US$ 181, sendo que 15,4% deste montante foram gastos com transporte e moradia e custos de acesso ao serviço de HIV. A despesa média informada pelos participantes para a consulta corrente ao centro de assistência de HIV foi de US$ 10. A despesa desembolsada apresentou regressividade, com os pacientes pertencentes à faixa de menor salário gastando proporcionalmente mais para obter atendimento. Estes resultados indicam a importância de considerar outros recursos necessários para obter acesso ao tratamento, como despesas de alojamento e o tempo necessário para ir a um centro de assistência de HIV e aguardar o atendimento por um profissional.

**Conclusões.:**

É preciso atentar mais às despesas incorridas diretamente pelas pessoas com HIV para que estes gastos não se convertam em barreiras ao acesso à assistência de saúde na República Dominicana. O design participativo baseado na comunidade do estudo permite que os dados sejam aproveitados para examinar as barreiras específicas ao atendimento de saúde enfrentadas por pessoas com HIV.

With an estimated adult human immunodeficiency virus (HIV) prevalence of 1.2% in 2017, the Caribbean is the second most affected region in the world after sub-Saharan Africa (1). Among Caribbean nations, Haiti has the highest number of persons with HIV, followed by the Dominican Republic (2).

Since the beginning of the HIV epidemic, the Dominican Republic has relied heavily on external funding to finance its HIV services. However, since international support is decreasing, the Dominican Government is expected to assume increasing financial responsibility for those services (3). According to the Joint United Nations Program on HIV/AIDS (UNAIDS), between 2016 and 2017, internationally sourced HIV expenditures in the Dominican Republic dropped from US$ 14.5 million to US$ 6.2 million, a decline from 54.3% to 29.7% of the country’s total HIV expenditures (4). In this context, most policy discussions have focused on finding new ways to sustain public HIV spending, to ensure that levels of prevention and treatment coverage are maintained (5).

This growing pressure to use domestic resources in the Dominican Republic, coupled with a health system supported mostly by direct payments made by households (6), creates a risk of increased out-of-pocket (OOP) expenditures to pay for access to HIV care. This could deter access and adherence to treatment and health care services for persons with HIV (7-13), in a country that already has important gaps in terms of antiretroviral therapy (ART) coverage (1).

Considering the potential impact of OOP expenditures on health and financial outcomes for individuals with HIV, policy discussions should take into account both public spending and private spending. However, the lack of information about the latter often hinders such discussions.

The aim of the study was to estimate OOP expenses incurred by people living with HIV (PLHIV) in the Dominican Republic and to analyze the characteristics of that spending for different groups of that population, especially across different wage ranges. Primary data was gathered using a community-based participatory research (CBPR) design. This data allowed us to develop a broader understanding of HIV care and treatment costs in the country, to be used as evidence to support national policy discussions as well as to serve as an advocacy tool for communities and individuals with HIV.

## METHODS

### Community-based participatory research design

The Dominican research project was implemented between July 2014 and June 2016, applying a community-based participatory research (CBPR) design. In CBPR, researchers partner with community members, bridging both worlds’ expertise through shared knowledge and capacity-building (14, 15).

The project was led by two advocacy groups, with oversight from a steering committee. The advocacy groups, AID FOR AIDS and the International Treatment Preparedness Coalition, work on access to HIV care in the Dominican Republic (among other countries). Members of both organizations were involved in the core project team, working in close collaboration with an experienced external researcher. All members of the core project team worked collaboratively to develop a survey for use among individuals living with HIV.

The steering committee was composed of community treatment activists as well as officials from United Nations agencies, the Pan American Health Organization (PAHO), and the Dominican Ministry of Health. The steering committee provided guidance regarding the study design and data collection.

Grassroots organizations, including networks of PLHIV, female and male sex workers, and transgender women, were invited to participate in a two-day workshop in May 2015. The first day of the workshop included a capacity-building component focused on how to conduct CBPR, as well as participatory sessions to review and validate the survey questionnaires and the data collection implementation strategy. After these meetings, small changes were introduced (use of colloquial language, additional response options, different ways of framing questions). The second day focused on training participants in survey methods, such as interview skills, research consent and ethics, and sampling selection strategy. The workshop was followed by a pilot study in a health center in the country’s capital, Santo Domingo.

All the participants who completed the entire workshop were invited to collaborate in the data collection process. The pilot study and the data collection process were supervised by two members of the AID FOR AIDS Dominican Republic office, who were present during the entire workshop and received additional training as supervisors. Approval for the study was obtained from the Dominican Republic’s National Council on Bioethics in Health (CONABIOS) on 10 March 2015.

### Survey design

In October and November of 2015, a cross-sectional survey was conducted among 191 individuals with HIV in the Dominican Republic. ART is currently free of cost in the 72 public HIV treatment and care units around the country (16), as well as in private pharmacies. For this study, 9 public and private nonprofit HIV treatment and care sites were selected in and around Santo Domingo. Private for-profit providers and pharmacies were excluded, given their limited role in HIV treatment in the country. Selected sites were purposively chosen to reflect different geographic locations and size (in terms of total number of HIV patients). Only sites serving more than 100 ART and pre-ART patients per year were included in the study.

At each site, an average of 20 individuals responded to a short interview (< 30 minutes). Participants were randomly selected out of all HIV patients who met the following criteria: a) have a confirmed HIV infection, b) be over 18 years old, and c) be currently enrolled in ART or pre-ART services or have been enrolled within the last six months prior to the interview.

Interviews were offered at the end of the individual’s visit at the HIV-care site. The trained community-member interviewers used printed questionnaires to interview study participants. Interviews were conducted in a private space to respect the participant’s confidentiality. In compensation for their time, survey respondents received 860 Dominican pesos (RD$ 860) (equal to US$ 18.90, as of November 2015). At the end of every day, the community-member interviewers entered the collected responses in an Excel database. A supervisor later cross-checked the database against the printed questionnaire, to eliminate any data entry mistakes.

### Questionnaire instrument

The survey questionnaire used with the PLHIV interviewees included a mix of multiple-choice and binary questions, as well as an open-ended comments section. The information collected focused on four themes: 1) basic sociodemographic characteristics, including gender, age, education, and health insurance status; 2) health status and general HIV services utilization, including the time since HIV diagnosis, current antiretroviral treatment, and reported adherence to treatment; 3) the current visit to the HIV facility, including its primary purpose and OOP expenditures for this visit; and 4) OOP expenditures associated with various expenditure categories related to HIV treatment and care in the preceding 6 months (except for inpatient HIV care expenditures, which were captured for the preceding 12 months; those inpatient expenditures were captured over 12 months in order to facilitate recall).

### Data analysis

All the data collected was merged, cleaned, and analyzed, using Stata software. The analysis included statistics on the mean OOP payments made by the survey respondents during the preceding six months and for the current visit; the distribution of OOP payments among expenditure categories; and wage ranges.

The study coauthors made several important choices during the analysis. One decision concerned the data collected, which allowed for an analysis of health expenditures for the current visit and the last six months. Both pieces of information are interesting, but they present a trade-off between accuracy (no recall bias) and representativeness of an individual’s “average” expenditure. Therefore, we used both pieces of information to present different calculations related to the financial burden of obtaining HIV treatment in the Dominican Republic.

Similarly, we coauthors made an important decision related to which categories of OOP expenditures should be included in our calculations. The World Health Organization (WHO) and the Organization for Economic Cooperation and Development (OECD) define OOP expenses as direct payments made by individuals where insurance does not cover the full cost of the health good or service, including cost-sharing, self-medication, and other outlays paid directly by private households (17, 18). For this study, OOP expenses for HIV services included drugs, health care services, lab tests, and medical supplies. However, based on discussions with advocates and community members, items such as transportation and lodging costs were also explicitly included in the questionnaire, because previous evidence and our CBPR design suggested these could be significant barriers to health care in the Dominican Republic (19). Consequently, results are presented for OOP health expenditures and for OOP health expenditures plus lodging and transportation, as a way to capture the “total cost” of receiving treatment, in line with models of spatial competition (20, 21).

Finally, although most calculations are presented at the aggregate level, others were categorized by wage ranges in order to explore potential income inequalities in paying for HIV treatment in the Dominican Republic.

One important limitation of the data is that neither income nor expenditures were collected as specific numbers; instead, ranges were used. One of the challenges with CBPR is resolving potential trade-offs between methodological choices to increase the study’s validity and incorporating the communities’ interests and concerns (22). In this case, an important role in the project—administering the survey questionnaire—was carried out by community-member interviewers. Since they were interviewing peers, information on income was considered sensitive, and wage ranges were used. This poses a challenge for expressing the OOP expenditures as percentages of either the total income or total expenditures for the individual. In order to overcome this problem, wage ranges are presented instead. All numbers are expressed in U.S. dollars as of November 2015, with the exchange rate of US$ 1.00 = RD$ 45.4 (23).

Written consent was obtained prior to all interviews. The consent form described the study goals, stated that participation was voluntary, and explained the strategy used by the research team to guarantee data anonymity.

## RESULTS

### Descriptive statistics

Descriptive statistics for our study are presented in Table 1. The sample was balanced between men and women, with a large majority of respondents classifying themselves as heterosexual. The sample also split equally between those who were married or living together and those who were single. The respondents’ mean age was 38.9 years. The sample was distributed closely among people with low (less than primary completed), medium (primary completed), and high (secondary completed or more) educational attainment.

**TABLE 1.** Descriptive statistics for participants in survey of out-of-pocket (OOP) expenditures for HIV in the Dominican Republic, 2015^a^

Variable	Statistic
Mean age in years (mean and standard deviation (SD))	38.9 (12.4)^b^
Gender (percent of the sample)	
Woman	55.8%
Man	41.1%
Transgender woman	3.2%
Sexual orientation (percent of the sample)	
Heterosexual	88.4%
Gay/Lesbian	9.1%
Bisexual	2.6%
Marital situation (percent of the sample)	
Single/never married	43.9%
Married or living together	41.3%
Other	14.7%
Schooling (percent of the sample)	
No formal schooling or less than primary school	38.6%
Completed primary school	33.3%
Completed secondary school or higher	28.0%
Work situation (percent of the sample)	
Employed	28.9%
Stay at home or student	27.4%
Looking for a job	25.3%
Other	18.4%
Health status and treatment	
Percent of the sample on antiretroviral (ARV) therapy	97.1%
Number of months on ARV treatment (mean and SD)	76.2 (125.3)
Number of months since diagnosis (mean and SD)	81.5 (59.5)
Number of comorbidities reported (mean and SD)	0.89 (1.2)
Health expenditure	
Percent of the sample with health insurance	53.4%
OOP expenditure for current visit (US$ Nov. 2015)^c^ (mean and SD)	US$ 3.6 (10.2)
OOP expenditure over last 6 months (US$ Nov. 2015)^c^ (mean and SD)	US$ 153.0 (279.3)
Economic situation	
Percent of the sample with monthly average individual wage < US$ 110	57.4%
Percent of the sample with monthly average household income < US$ 440	75.6%
Distance in kilometers to health center (mean and SD)	19.7 (41.9)
Number of children (mean and SD)	2 (1.8)
Spanish is the main household language (percent of the sample)	97.9%

***Source:*** Prepared by authors, based on the study results.

a All statistics presented in this table include responses from between 188 and 191 individuals out of the 191 individuals interviewed for the study, except for the category of “mean OOP expenditure over the last 6 months,” which is reported for 182 individuals.

b In this table, standard deviation (SD) values are presented in parentheses.

c In November 2015, the exchange rate was US$ 1.00 = RD$ 45.4 (23).

Study participants had been diagnosed with HIV for a mean of 7 years and had started treatment a mean of 6 years prior to the interview. In terms of antiretroviral treatment, 97.1% of respondents said they were receiving antiretroviral treatment at the time of the interview. The average number of comorbidities reported in the sample was 0.9. Half of the interviewees said they had health insurance.

Almost 60% of the respondents said they had a mean monthly wage of US$ 110 (RD$ 5 000) or less, while 29.8% of the sample said they had no wages at all. These earnings are low compared to the average monthly income per capita of US$ 338, as calculated by the National Statistics Office (ONE) (24). More than 75% of the sample reported living in a household with an average income of US$ 442 or less. Finally, people reported traveling an average of 20 kilometers to access their HIV center.

### Overview of out-of-pocket expenditures for the preceding six months and the current visit

Study participants reported mean HIV health-related expenditures of US$ 153.0 during the preceding six months (about US$ 26 per month), and mean OOP expenditures for the current visit of US$ 3.6. In comparison, according to the 2013 Demographic and Health Survey (DHS) for the Dominican Republic (25), the inflation-adjusted average monthly health expenditure by Dominican households overall was US$ 15 (RD$ 682) when considering every household in the sample and US$ 27 (RD$ 1 221) when taking into account only those with nonzero expenditures. The numbers include expenditures for medical visits, drugs, tests, procedures, and any hospital stay.

Expenditures from our survey were large, considering they included only HIV-related costs and were only based on individual expenditures, while the Dominican DHS presented statistics at the household level. When including other costs incurred in obtaining treatment (transportation and housing), expenditures reported by the survey participants for the preceding six months increased to a mean of US$ 181 and OOP expenditures for the last visit reached a mean of US$ 10. The large fraction attributable to transportation and housing in the last visit is mostly explained by the fact that most people reported positive expenditures for some of these items, while the percentage of the sample reporting positive expenditures for other items was lower.

Table 2 shows that survey participants with no wages reported mean HIV-related OOP expenditures of US$ 29. Expenditures generally grew with wages. It is important to note again the difference between using individual wages versus household income. The people reporting no wages still had expenditures. That meant that despite having no wages, the participants still had a source of funds (e.g., assets, other household member’s income) that allowed them to pay for OOP expenditures. However, when looking at the share of wages devoted to HIV spending, OOP payments appear to be regressive. That is, people with lower wages used a larger fraction of those funds to pay for their HIV treatment. This regressivity was also found when looking at OOP payments as a proportion of total expenditures.

**TABLE 2:** HIV-related out-of-pocket (OOP) expenditures (US$, November 2015) as a share of the total wage of survey participants, grouped by socioeconomic level, Dominican Republic, 2015^a^

		Health expenditure only				Health expenditure + transportation and housing		
Wage range (US$)		Monthly spending (US$)		Range OOP spending/wages (%)b	OOP spending/wages (%)^c^	Monthly spending (US$)	Range OOP spending/wages (%)^b^	OOP spending/wages (%)^c^
		Min.	Max.			Min.	Max.	
No wages	29.1	NA	NA	NA	33.3	NA	NA	NA
1 to 110	16.9	15.4%	NA	30.8%	21.8	19.8%	NA	39.6%
Between 110 and 220	21.1	9.6%	19.2%	12.8%	25.4	11.5%	23.1%	15.4%
Between 220 and 440	46.7	10.6%	21.2%	14.1%	53.4	12.1%	24.2%	16.2%
440 and more	44.8	NA	10.2%	10.2%	49.9	NA	11.3%	11.3%

***Source:*** Prepared by authors, based on study results.

a In November 2015, the exchange rate was US$ 1.00 = RD$ 45.4 (23).

b The “Range OOP spending/wages” columns’ minimum and maximum values were calculated as the average monthly HIV-related spending (cumulative six-month spending divided by six) divided by the lowest/highest value of the wage range. For example, for the “between 110 and 220” row of the “health expenditure only” column, the range’s lower limit was calculated as 21.1/220 = 0.96 = 9.6%, whereas the upper limit was calculated as 21.1/110 = 0.192 = 19.2%. The “NA” [not applicable] indicates where estimates were meaningless, since the denominator was either zero or infinite.

c The OOP spending/wages values were calculated as the average monthly HIV-related spending (cumulative six-month spending divided by six) divided by the midrange value of the wage range. For example, for the “between 110 and 220” row of the “health expenditure only” column, the value was calculated as 21.1/([110+220/2]) = 0.128 = 12.8%.

The numbers reported for our survey had limitations, including the use of individual wage ranges instead of household income. Therefore, the numbers should be interpreted with caution. However, the estimates are useful, since they provide an order of magnitude and reveal an interesting trend.

A similar pattern exists when comparing our results with national health expenditure data from the DHS survey. The DHS survey found that Dominican families’ health expenditures as a share of income decreased as income rose, with the share being 35.9% for households with an income lower than US$ 110 (RD$ 5 000) and about 13.0% for higher income categories (25). The results were similar when considering households’ transportation and housing expenses. Adding these costs increased the estimate of the share of wages devoted to obtaining HIV treatment, but more importantly, also increased the regressivity. For people earning over US$ 110, the additional costs entailed an increase of about 2 percentage points in the ratio of OOP expenditures to wages. For those who made less than US$ 110, these costs produced an increase of almost 10 percentage points.

Finally, just as nonhealth costs proved to be important when accounting for the total cost of receiving HIV care in the Dominican Republic, nonmonetary costs should also be considered. When discussing access to treatment, an important factor to bear in mind is time. No clear relationships emerged between wage ranges and time spent traveling to and waiting at the HIV facility. However, all groups reported waiting and traveling times close to three hours, representing a significant loss of productivity, especially for those with lower wages (Table 3).

**TABLE 3:** Average time (hours) spent by survey participants in obtaining treatment at last visit to the health center, by wage ranges, in study of out-of-pocket expenditures for HIV, Dominican Republic, 2015

Wage range (US$)^a^	Travel time	Waiting time at health center	Total time spent during the last visit to the health center
No wages	1.05	1.79	2.85
1 to 110	1.03	1.68	2.71
Between 110 and 220	1.00	1.42	2.42
Between 220 and 440	1.16	1.69	2.85
440 and more	1.37	1.24	2.61

***Source:*** Prepared by authors, based on study results.

a In November 2015, the exchange rate was US$ 1.00 = RD$ 45.4 (23).

### Distribution of out-of-pocket expenditures in the preceding six months

**FIGURE 1:**
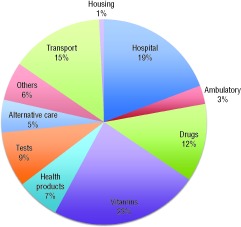
Distribution of out-of-pocket expenditures across different HIV-related services in the preceding six months among survey participants, expressed as a percentage, Dominican Republic, 2015.

***Source:*** Prepared by authors, based on the study results.

Figure 1 shows the distribution of HIV-related expenditures for the preceding six months. As discussed before, using the expenditures from the last visit is complex since several respondents reported zero expenditures in many categories. The leading expenditure category was drugs and vitamins (totaling 35.7%), which was similar to results found in studies of OOP expenses in South American countries (26). Together, housing and transportation represented 15.4% of the total expenditure, which was more than the costs of some other expenses, such as drugs and tests.

Table 4 shows that the distribution of OOP payments by expenditure category differs among people with different wages. In terms of the percentage of their total HIV-related OOP expenses, higher-wage patients spend proportionally more on inpatient and outpatient care, while those with lower wages spend proportionally more on health products and transportation.

**TABLE 4:** Distribution of HIV-related out-of-pocket (OOP) expenditures made in the last six months by survey participants, grouped by wage ranges, as a percentage of total OOP expenditures, Dominican Republic, 2015

Wage range (US$)^a^	Hospital	Ambulatory	Drugs	Vitamins	Tests	Health products	Alternative care	Other	Transportation	Housing
No wages	19.2	0.9	14.2	26.0	8.1	7.2	5.8	6.2	10.7	1.9
1 to 110	17.9	2.0	8.0	18.5	9.7	15.6	3.7	3.9	20.4	0.3
Between 110 and 220	9.2	1.3	10.9	28.6	10.2	3.3	9.5	9.9	16.6	0.5
Between 220 and 440	31.9	9.4	11.7	21.7	7.7	2.2	1.4	1.4	12.4	0.0
More than 440	32.4	4.4	28.0	8.7	3.6	0.0	0.0	12.8	10.2	0.0

***Source:*** Prepared by authors, based on study results.

a In November 2015, the exchange rate was US$ 1.00 = RD$ 45.4 (23).

## DISCUSSION

To our knowledge, this study is the first ever to collect such detailed information regarding patterns of private spending associated with HIV care and treatment in the Dominican Republic. Its findings provide a better understanding of the sociodemographic characteristics of people with HIV currently enrolled in care, as well as the level and distribution of their OOP expenditures.

It is also important to note that the study proposes a different approach—community-based participatory research—that can be used to draw lessons about the benefits and challenges of involving and empowering communities in research. We hope this experience will encourage more researchers to work for and with communities in the future.

The Dominican Government tries to provide HIV treatment free of charge. Nevertheless, our study reveals that PLHIV in the country still spend a large amount of money on items related to their HIV treatment, such as nutritional supplements and transportation to and from health facilities. Furthermore, these additional expenditures are not evenly spread across our sample. The regressivity of the OOP HIV expenditures shows the importance of focusing on barriers to access and of considering social needs and the social determinants of health in health care costs, particularly when thinking of low-income patients.

These findings are relevant for two key reasons. First, the results highlight the need to adopt a holistic approach to HIV expenditures when discussing shifts in HIV funding for countries of Latin America and the Caribbean. The National AIDS Spending Assessment (NASA), an HIV resource-tracking tool developed by UNAIDS, does not include private OOP expenditures in its assessment of resources devoted to HIV. As a result, most studies of Latin American and the Caribbean countries that draw on NASA data do not include this expenditure category in their analyses (27, 28). Furthermore, such data is often not even collected by national authorities due to the lack of resources, leading OOP outlays to be “invisible” in national policy discussions. Our results show the relevance of considering these items in order to capture the “full cost” of receiving treatment. Similarly, it is also important to bear in mind the opportunity cost of obtaining treatment—as measured in terms of time spent—as a potential factor when thinking about barriers in access to health care treatment.

Second, our results show the high heterogeneity found in OOP expenditures among individuals with HIV in the Dominican Republic. The study clearly indicates that some groups are more vulnerable than others to incurring high levels of expenditures when accessing HIV treatment. Such granular analysis can provide insights beyond health spending, such as reasons for lack of adherence and retention. Since these findings were developed in close collaboration with grassroots organizations in the Dominican Republic, they can be used by both policymakers and communities themselves to design programs and initiatives specifically targeted to addressing these additional barriers to care, such as by developing transportation voucher programs or additional financial support for nutritional treatment, as is being done in many countries.

## Limitations

This study had several limitations. First, there was a potential for recall bias, especially with regard to expenditures in the preceding six months. This limitation was partially addressed by using two types of spending—current visit and last six months—to capture different dimensions of HIV-related expenditures.

A second limitation was the lack of information on income and expenditure. Using wage ranges allowed some descriptive analysis but limited other analysis to better understand the sources of differences in OOP spending among different groups in the country. As stated before, results must be interpreted with caution—particularly when comparing them with other estimates—given the differences arising from the unit of analysis, individual wage versus household income, especially when looking at the results for participants with no wages.

Third, due to budget and logistic constraints, the number of facilities selected was limited and their selection made purposively. Consequently, the results are not statistically representative of the Dominican reality. However, the information presented is valuable as it gives, for the first time, an estimate of the financial burden faced by PLHIV in the country.

Finally, our sample only includes individuals with HIV already accessing HIV treatment and care. In 2017, UNAIDS estimated that 68% of individuals with known HIV were then on ART (while only 77% of all people with HIV knew their status) (4). For the remaining 32%, it is unclear if HIV expenditures play a role in not accessing health care. Consequently, these results might underestimate the impact of financial barriers in accessing HIV care in the Dominican Republic.

## Conclusions

This study has shown that OOP expenditures still represent a significant burden for PLHIV in the Dominican Republic, particularly those with lower wages. Regressivity in OOP spending should be considered when debating access to care. This is particularly true, given that some other costs—both monetary (e.g., transportation and lodging) and nonmonetary (e.g., time)—are unevenly distributed among participants. Ensuring that such expenses are part of an open, national discussion can ultimately guarantee effective access to HIV care and treatment.

This study is a concrete example of the use of CBPR in public health research. It shows that while community involvement can constrain the research process, it also presents clear opportunities for co-learning. Using CBPR can also facilitate the use of financial and cost data for advocacy purposes, as it facilitates research ownership by community-based organizations. Finally, and most importantly, CBPR can ensure that the voices and priorities of those most directly affected by changes in HIV funding can be heard.

## Author contributions.

CC and CO conceived the original idea. CC, CO, EC, and JV participated in the study design and implementation. CC and PVD performed the analysis and wrote the first draft. All the authors reviewed and approved the final version.

## Acknowledgments.

The authors want to thank Sergio Bautista Arredondo for his comments on the study design and survey instrument. We are grateful to all the workshop participants for their valuable feedback on the study design and tools and their involvement during data collection, as well as to Virginia Moreno and Kiara Mariano, from the AID FOR AIDS Dominican Republic office, for their help coordinating the data collection process. Bill Black and Sadiya Muqueeth provided valuable suggestions for improving the manuscript. We also appreciate the comments and suggestions given by three anonymous reviewers and an editor for the *RPSP/PAJPH*.

## Disclaimer.

The authors hold sole responsibility for the views expressed in the manuscript, which may not necessarily reflect the opinion or policy of the *RPSP/PAJPH* and/or PAHO.
